# Hybrid method for automatic initialization and segmentation of ventricular on large-scale cardiovascular magnetic resonance images

**DOI:** 10.1186/s12880-025-01683-4

**Published:** 2025-05-07

**Authors:** Ning Pan, Zhi Li, Cailu Xu, Junfeng Gao, Huaifei Hu

**Affiliations:** 1 College of Biomedical Engineering, South-Central Minzu University, Wuhan, 430074 China; 2 Hubei Key Laboratory of Medical Information Analysis and Tumor Diagnosis & Treatment, Wuhan, 430074 China; 3 Key Laboratory of Cognitive Science, State Ethnic Affairs Commission, Wuhan, 430074 China

**Keywords:** Cardiac image analysis, Deep learning, Statistical shape models, Complex transformation

## Abstract

**Background:**

Cardiovascular diseases are the number one cause of death globally, making cardiac magnetic resonance image segmentation a popular research topic. Existing schemas relying on manual user interaction or semi-automatic segmentation are infeasible when dealing thousands of cardiac MRI studies. Thus, we proposed a full automatic and robust algorithm for large-scale cardiac MRI segmentation by combining the advantages of deep learning localization and 3D-ASM restriction.

**Material and methods:**

The proposed method comprises several key techniques: 1) a hybrid network integrating CNNs and Transformer as a encoder with the EFG (Edge feature guidance) module (named as CTr-HNs) to localize the target regions of the cardiac on MRI images, 2) initial shape acquisition by alignment of coarse segmentation contours to the initial surface model of 3D-ASM, 3) refinement of the initial shape to cover all slices of MRI in the short axis by complex transformation. The datasets used are from the UK BioBank and the CAP (Cardiac Atlas Project). In cardiac coarse segmentation experiments on MR images, Dice coefficients (Dice), mean contour distances (MCD), and mean Hausdorff distances (HD95) are used to evaluate segmentation performance. In SPASM experiments, Point-to-surface (P2S) distances, Dice score are compared between automatic results and ground truth.

**Results:**

The CTr-HNs from our proposed method achieves Dice coefficients (Dice), mean contour distances (MCD), and mean Hausdorff distances (HD95) of 0.95, 0.10 and 1.54 for the LV segmentation respectively, 0.88, 0.13 and 1.94 for the LV myocardium segmentation, and 0.91, 0.24 and 3.25 for the RV segmentation. The overall P2S errors from our proposed schema is 1.45 mm. For endocardium and epicardium, the Dice scores are 0.87 and 0.91 respectively.

**Conclusions:**

Our experimental results show that the proposed schema can automatically analyze large-scale quantification from population cardiac images with robustness and accuracy.

## Introduction

Exponential growth of cardiac data is due to continuous progress in biomedical devices and technologies, which creates an opportunity for exploring the underlying mechanisms of disease, as well as a challenge for current capabilities to extract objective and quantitative cardiac phenotypes. Being the leading cause of death worldwide [1], cardiovascular diseases are an important societal health concern and burden.

Quantitative analysis of cardiac function requires establishing global or regional parameters of cardiac performance such as: left ventricular End-diastolic Volume (LVEDV) and left ventricular End-systolic Volume (LVEDV) for the blood pool, left ventricular mass (LVM) of the myocardium, left ventricular ejection fraction (LVEF), left ventricular stroke volume (LVSV), and wall thickening or wall thinning. To compute any of these parameters, the left ventricle must be segmented. However, it is tedious and time consuming task for cardiologists, radiologists or technicians to manually, or semi-manually (aided by software) identify and delineate the relevant cardiac structures for further analysis. Inter and intra-observer variability also undermines the validity of the derived parameters. Therefore, methods are desperately needed to accelerate and facilitate the process of image segmentation to support diagnosis, treatment evaluation and patient follow-up. A number of algorithms have been proposed for automatic and semi-automatic cardiac MRI (CMR) segmentation: image-based [2–4], pixel-/voxel-level classification [5], deformable models [6–9], atlas construction [10], and machine learning [11–13]. For a detailed account of previous work we refer the reader to recent topical reviews [14–16].

However, most of the above algorithms cannot meet the needs faced when dealing with large-scale heterogeneous populations. To address these scenarios, a robust technique known as ASM (active shape model) [17] can be employed for visualizing and quantifying both geometric and functional patterns of the heart. This method leverages prior knowledge by encoding the distinct shape and appearance variations present in the images. When the shape models are adopted for segmentation, the 3D-ASM surface needs to be initialized within the capture range of the intended boundaries for a robust and accurate fit. To get the initial shape for a single patient, Catalina et al. adopted a simple mechanism to roughly scale and position the mean shape of the model [18]. Three points are manually selected, two epicardial points at the basal level, and a third one at the apex. Corresponding anatomical landmarks of the mean shape were previously defined by an experienced operator. Using a similarity transformation, the initial shape can be derived after the mean shape is aligned to the landmarks. However, manual initialization becomes infeasible when dealing with thousands of CMR volumes. Xènia et al. proposed a fully automatic method for initializing cardiac MRI segmentation, by using image features and random forests regression to predict an initial position of the heart and key anatomical landmarks in an MRI volume [19]. However, this method relies on the intersection of the two LA (long axis) and the SA (short axis) images. This may result in failure when intersections cannot be obtained from the needed images. In addition, initial shapes relying on landmark detection sometimes cannot cover all slice images, especially at the basal and apex levels.

Currently, deep learning techniques have been widely used by scholars in pattern recognition, computer vision and medical image computing [20–24]. Avendi et al. adopted deep convolutional neural networks (CNNs) to locate the LV, then inferred the LV shape using stacked auto encoders [25]. Excellent agreement with the ground truth was achieved for the endocardial contours using datasets from MICCAI 2009 LV segmentation challenge [3]. Inspired by combining their successful methods with the advantages of a model-based approach, here we investigate the analysis of a large population of images using both CNNs and SSMs. We design a new schema to build the initial shape for 3D-ASM, then apply the 3D-ASM method to introduce high-level knowledge on cardiac anatomy and deal with sparsely distributed multi-view slices in CMR. Our 3D-ASM implementation is aided by knowledge on the rough localization of the myocardial boundaries, produced by CNNs, to produce a stable and robust delineation of both endo and epicardial surfaces. Therefore, we propose a cardiac segmentation framework that, unlike some approaches requiring preprocessing operations on image data such as denoising and enhancement [26], is an end-to-end segmentation method based on CNNs, which streamlines the process by removing traditional preprocessing steps. Then, by combining CNNs with SSMs, this method features better localization and morphological accuracy in LV segmentation.

Our paper is organized as follows. In the next section, we detail our pipeline for cardiac image segmentation. Then data source used in this study is described. In the following results section, extensive comparisons are made to show the properties of our methods. Finally, we discuss the significance of our work.

## Method

### Overview

**Fig. 1:**
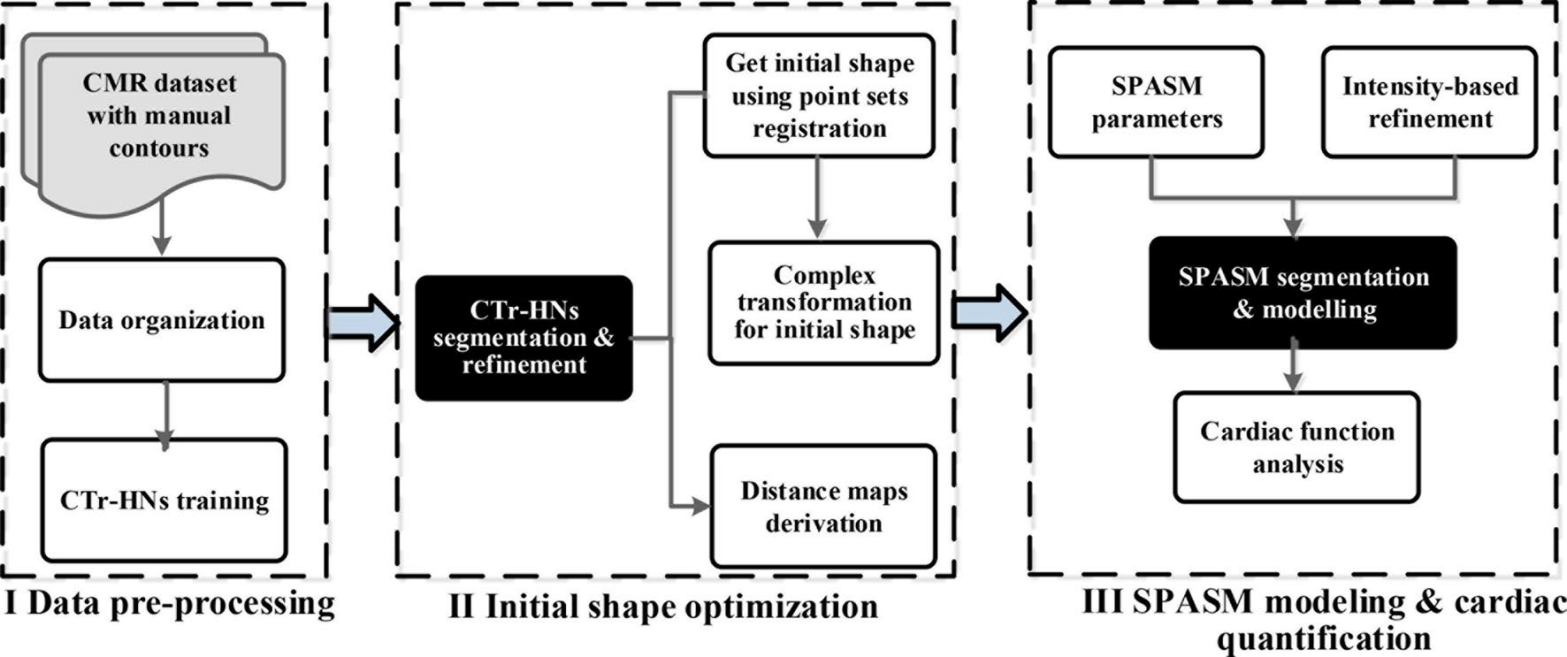
Pipeline for the proposed cardiac magnetic resonance segmentation

In this section, our work-flow exploits automatic initialization and segmentation of the left ventricle using 3D-ASM. Here the statistical shape model used is SPASM (sparse active shape model) [27]. Our algorithm includes three steps, i.e. Data pre-processing, Initial shape optimization and SPASM modeling & cardiac quantification, as depicted in Fig. 1. In the beginning, cardiac MR datasets with ground truth are organized according to the time frames per subject, CTr-HNs (integrated CNNs and Transformer for heart segmentation networks) is applied to train these organized cases. Secondly, the test cases are sent to CTr-HNs to get segmentation. As a result, the masks for endo- and epi-cardial can be derived separately. Consider that CTr-HNs may cause some bad segmentation, the masks from CTr-HNs are refined subsequently. Then the mean Point Distribution Model (PDM) is fit to the endo- and epi-cardial points from CTr-HNs using point sets registration [28] to get an initial shape and the initial shape are refined using complex transformation subsequently. Distance maps are computed from the endo and epicardial walls obtained by CTr-HNs, which are subsequently used to drive the SPASM model towards image boundaries. Thirdly, SPASM is applied to refine the fit of the static shape model to the image data while penalizing large deviations from the ground truth, and the obtained results are employed for cardiac function analysis.

### Initialization of SPASM

#### Cardiac localization and segmentation

**Fig. 2:**
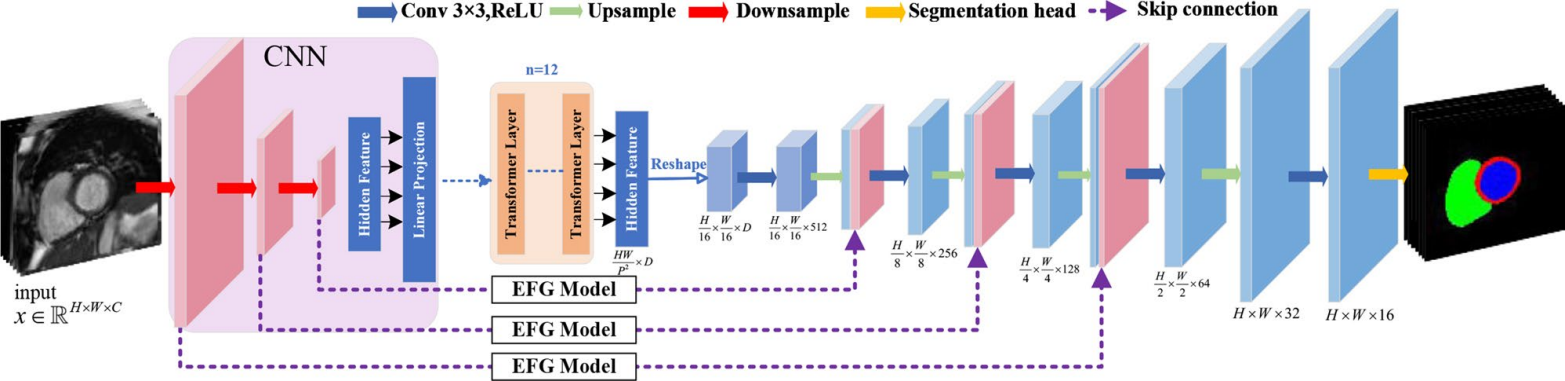
The architecture of the fully neural network

In our task of cardiac segmentation, we adopt a hybrid network integrating CNNs and Transformer [29] as a hybrid encoder for the segmentation of cardiac on MRI images. This architecture is named as CTr-HNs. An overview of the network architecture can be seen in Fig. 2.

**Fig. 3:**
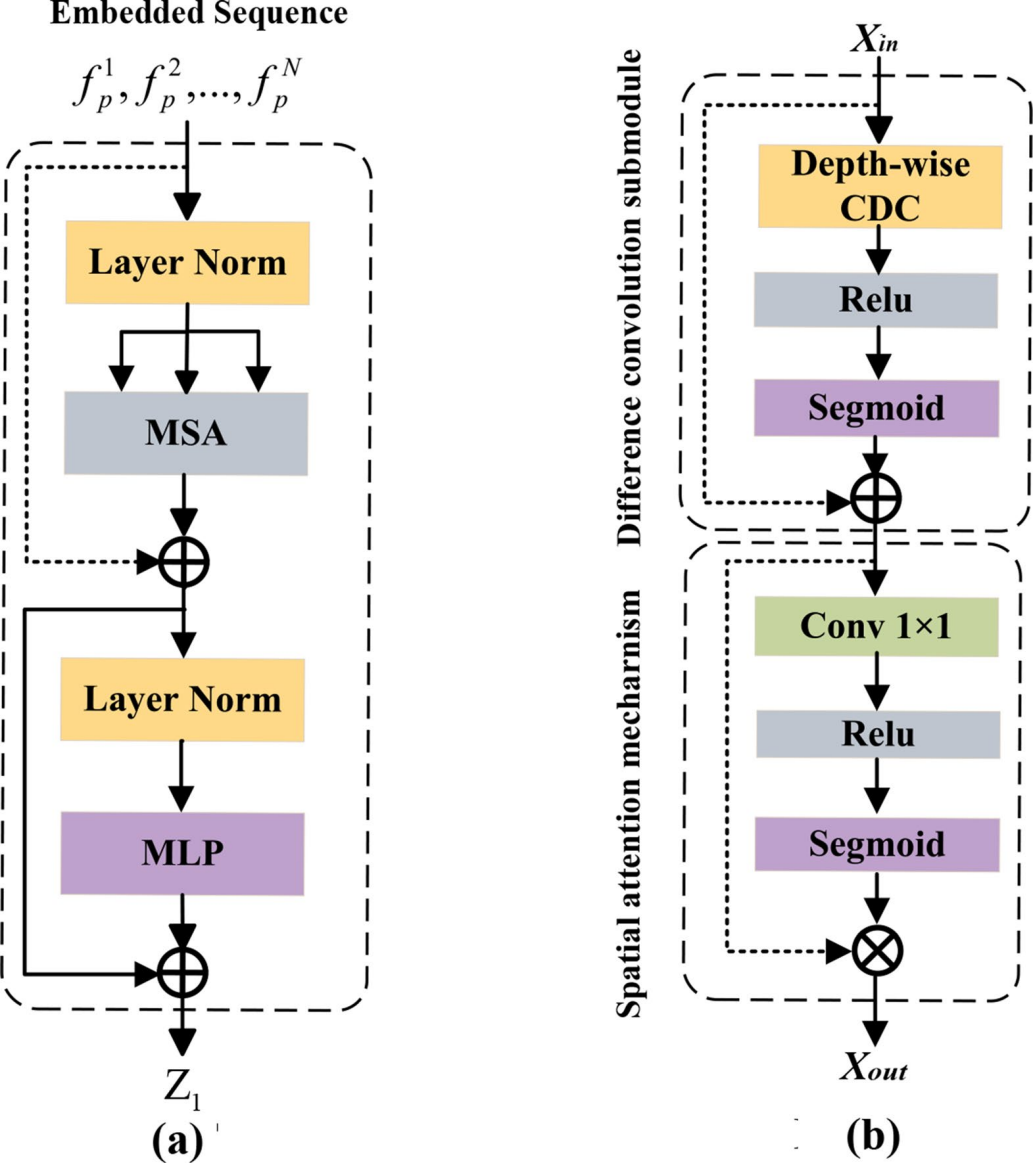
Transformer Layer and EFG Module. (**a**) Transformer Layer; (**b**) EFG Module

Given an image $x \in {\mathbb{R}^{H \times W \times C}}$ with spatial resolution of $H \times W$ and C-channels, the objective is to generate a prediction of the corresponding pixel-level labeled map with the size $H \times W$. Initially, the CNNs process MRI image to capture the local features. These features include details of edge, texture, and spatial information, which are progressively generated through convolutional and pooling operations to form multi-scale feature maps. Subsequently, the feature map is partitioned into $\{ f_p^i \in {\mathbb{R}^{{P^2} \cdot {\text{C}}}}|i = 1,\ldots,N\}$ by a patch serialization operation, where each patch has a size of $P \times P$ and the number of image patches is $N = \frac{{HW}}{{{P^2}}}$. Each patch is subsequently projected into a D-dimensional embedding space using a trainable linear transformation. Additionally, the spatial position information of each patches is encoded to obtain an embedding sequence of $f_p^i = [f_p^1,f_p^2,\ldots,f_p^N|i = 1,2,\ldots,N]$, where the sequence dimension is ${f_p} \in {\mathbb{R}^{\frac{{HW}}{{{P^2}}} \times D}}$. Then this sequence is fed into the 12 Transformer Layers. One-layer Transformer structure consists of Multi-head Self-Attention Mechanism (MSA) and Multi-Layer Perceptron (MLP) blocks (See Fig. 3(a)). The Transformer effectively compensates for the limited receptive field of CNNs, generating features with global dependencies, thereby providing rich contextual information for the subsequent decoder.

After the hybrid encoder, we can obtain the sequence $Z_L^i = [Z_L^1,Z_L^2,\ldots,Z_L^N|i = 1,2,\ldots,N]$ with the size of $Z_L^{} \in {\mathbb{R}^{\frac{{HW}}{{{P^2}}} \times D}}$. The sequence hidden features $Z_L^i$ are fed into the bottleneck layer. To restore the spatial order of the sequence, the encoded features are reshaped from $\frac{{HW}}{{P{}^2}} \times D$ to $\frac{H}{P} \times \frac{W}{P} \times D$ to match the input requirements of the subsequent decoder. In the decoder component, a cascaded structure of up-sampling and convolution operations is employed to progressively recover the resolution. Each level consists of 2 upsampling operations, one $3 \times 3$ convolutional layer, and one ReLU layer, progressively restoring the feature map from size $\frac{H}{P} \times \frac{W}{P}$ to the original resolution $H \times W$.

Additionally, the feature maps ${X_{in}}$ obtained through upsampling are concatenated with the feature maps from the CNNs through the EFG (Edge feature guidance) module [30] along the channel dimension to achieve feature fusion. The skip connections combine high-resolution local features with global contextual information, and the EFG module further enhances edge features ${X_{out}}$, ultimately predicting the segmentation labels. The structure of the EFG module consists of a difference convolution operator and a spatial attention mechanism (See Fig. 3(b)). The difference operation extracts edge information from the image, while the spatial attention mechanism enhances the feature representation of edge regions, guiding the network to better localize and segment the target area, effectively avoiding the issue of blurred boundaries in traditional networks.

During the training process, the loss function of CTr-HNs is the sum of The Cross-Entropy (CE) loss and the Dice loss, as shown as follows:

1 $$Los{s_{total}} = Los{s_{ce}} + Los{s_{dice}}$$

To balance the CE loss and Dice loss, the final loss function is the weighted sum of CE and Dice loss, as shown in Eq. (2), the weights ${w_1}$ and ${w_1}$ are learnable parameters and subject to ${w_1} + {w_2} = 1$.

2 $$Los{s_{total}} = {w_1}Los{s_{ce}} + {w_2}Los{s_{dice}}$$

All coarse segmentation experiments are run on NVIDIA RTX A5000 GPU with 24GB RAM. CTr-HNs are trained for 300 epochs with a batch size of 6, and the Adam optimizer, with an initial learning rate of 1e^−4^ and the weight decay constant of 3e-5, is used to iteratively update all parameters in the network. During training, the cosine annealing schedule to select the optimal learning rate. Additionally, to improve the robustness of CTr-HNs, in pre-processing, we also performed data augmentation operations on the training dataset, including rotation, translation, horizontal flipping, and vertical flipping.

To optimize initialization for SPASM, a slice-by-slice evaluation of the CTr-HNs segmentation starts from mid-slice and extends to the top-end slice and the bottom-end slice separately. For a slice image, if the CTr-HNs fails to process it, then the CTr-HNs results from neighbor slice are assigned to those of the current slice. Prior information about spatial relationships between slice segmentation is considered in this process, which makes the initialization accurate and robust.

**Fig. 4:**
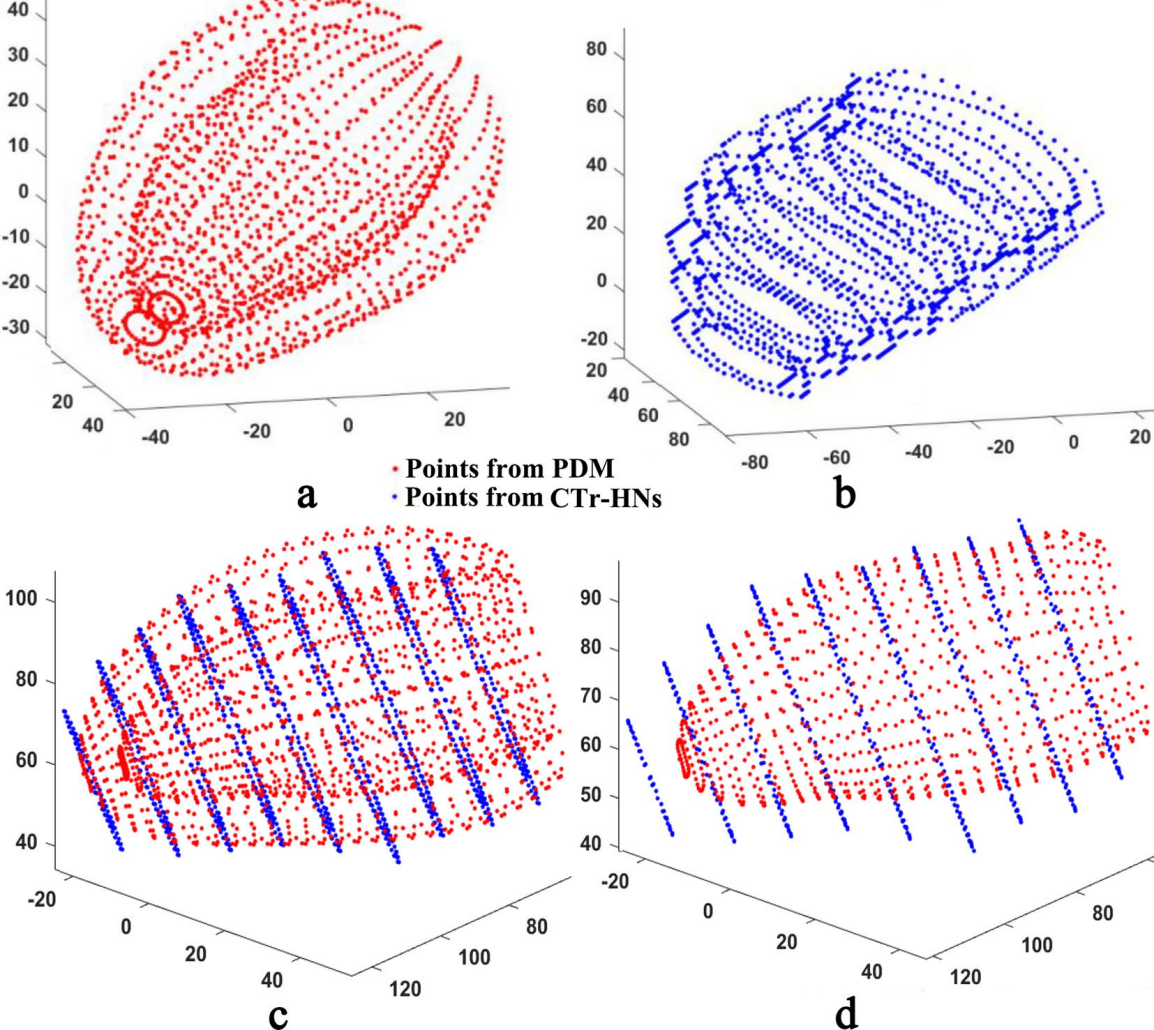
SPASM initialization. (**a**) Points from the mean PDM; (**b**) Points from CTr-HNs outputs; (**c**) Approximation of PDM to the points derived from CTr-HNs results; (**d**) Endocardial point sets derived from registered initial shape and CTr-HNs results respectively

Figure 4 shows the matching process for the initial shape of the SPASM. In Fig. 4(c), the initial shape is derived using a point-set registration algorithm [31]. However, the matching result is not optimal since the initial shape cannot cover all slices, which can be seen in Fig. 4(d). It is necessary to develop a technique to optimize the initial shape for SPASM. This refinement will be detailed in next step.

#### Initial shape refinement

Let’s assume a points set $P$ with ${\text{n}}$ points each described by three-dimensional coordinates ${{\text{p}}_i}({x_i},{y_i},{z_i})$ with $i = 1 \ldots {\text{n}}$. Assume $\overline P {\text{(}}\overline {\text{x}} {\text{ }}\overline {\text{y}} {\text{ }}\overline {\text{z}} {\text{)}}$ is the center of points set $P$.

3 $$\left\{ {\begin{array}{*{20}{c}} {\overline {\text{x}} = \frac{1}{n}\sum\limits_{i = 1}^n {{{\text{x}}_i}} } \\ {\overline {\text{y}} = \frac{1}{n}\sum\limits_{i = 1}^n {{{\text{y}}_i}} } \\ {\overline {\text{z}} = \frac{1}{n}\sum\limits_{i = 1}^n {{{\text{z}}_i}} } \end{array}} \right.$$

Hence, the matrix ${\text{X}}$ is

4 $$X = \left[ {\matrix{ {{{\rm{x}}_1}{\rm{ - }}\overline {\rm{x}} } & {{{\rm{y}}_1}{\rm{ - }}\overline {\rm{y}} } & {{{\rm{z}}_1}{\rm{ - }}\overline {\rm{z}} } \cr {...} & {...} & {...} \cr {{{\rm{x}}_i}{\rm{ - }}\overline {\rm{x}} } & {{{\rm{y}}_i}{\rm{ - }}\overline {\rm{y}} } & {{{\rm{z}}_i}{\rm{ - }}\overline {\rm{z}} } \cr {...} & {...} & {...} \cr {{{\rm{x}}_n}{\rm{ - }}\overline {\rm{x}} } & {{{\rm{y}}_n}{\rm{ - }}\overline {\rm{y}} } & {{{\rm{z}}_n}{\rm{ - }}\overline {\rm{z}} } \cr } } \right]$$

**Fig. 5:**
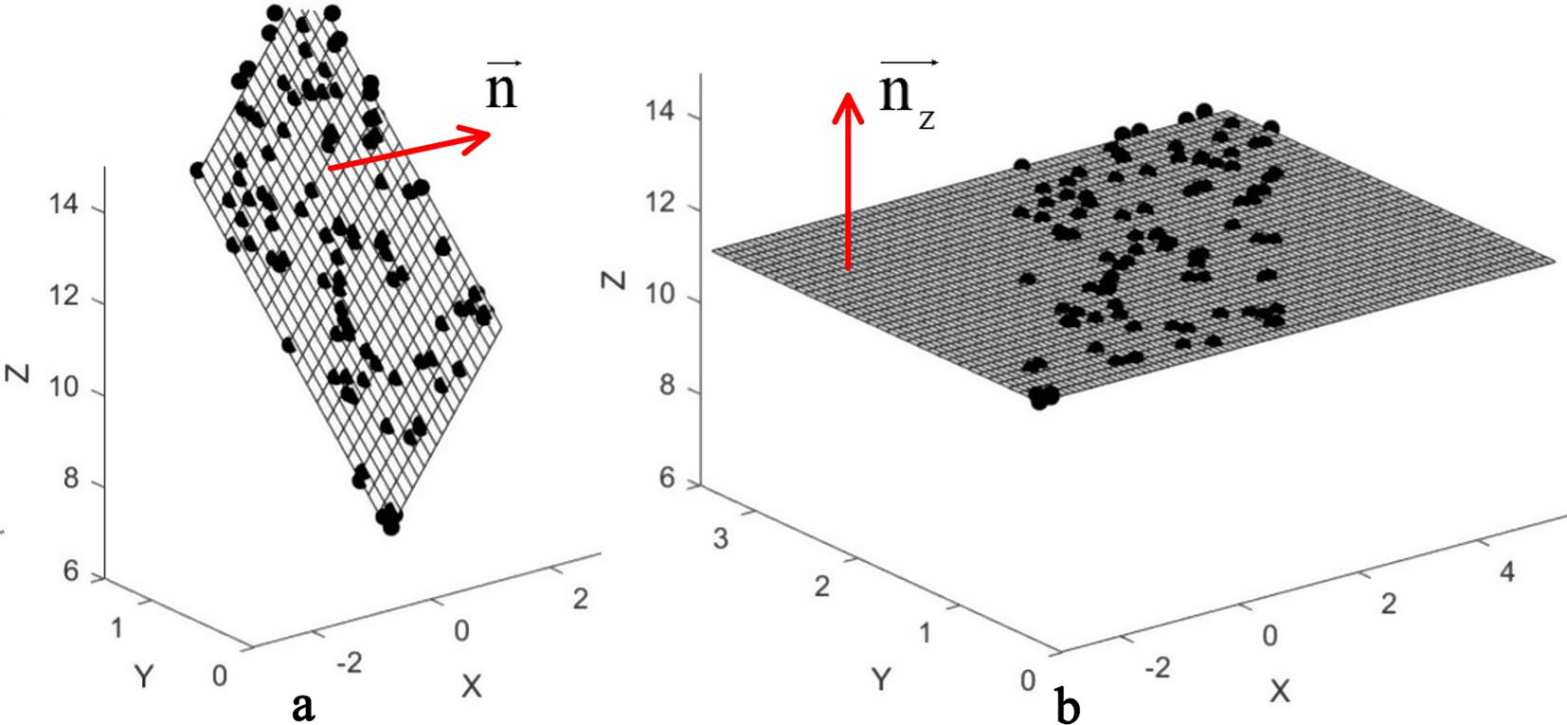
Plane fitting of points set and complex transformation. (**a**) Plane fitting from 3D points set; (**b**) Rotating the fitting plane perpendicular to Z-axis around the center point of the 3D points set

Singular value decomposition is applied to ${\text{X}}$ producing a diagonal matrix S, of the same dimension as X and with nonnegative diagonal elements in decreasing order, and unitary matrices ${\text{U}}$ and ${\text{V}}$ so that

5 $$X{\text{ }} = {\text{ }}U*S*V'$$

where ${\text{V = }}\left( {{{\text{v}}_1},{{\text{v}}_2}{\text{,}}{{\text{v}}_3}} \right)$, and ${{\text{v}}_3}$ is corresponding to the smallest singular value. A fitting plane $P{\text{l}}$ passing through the center point $\overline P {\text{(}}\overline {\text{x}} {\text{ }}\overline {\text{y}} {\text{ }}\overline {\text{z}} {\text{)}}$ can be obtained with unit normal vector $\overrightarrow n$ (See Fig. 5(a)).

6 $$\left\{ {\begin{array}{*{20}{c}} {\overrightarrow n {\text{ }} = {\text{ (cos}}\alpha {\text{ cos}}\beta {\text{ cos}}\gamma {\text{)}}} \\ {\overrightarrow {{n_z}} {\text{ }} = {\text{ (}}0{\text{ 0 1)}}} \end{array}} \right.$$

Where $\cos \,\alpha$, ${\rm{cos}}\,\beta$ and ${\rm{cos}}\,\gamma$ are directional cosines with x-, y- and z-axes respectively, is Z-axis unit normal vector.

Then the fitting plane $P{\text{l}}$ is rotated around the center point $\overline P {\text{(}}\overline {\text{x}} {\text{ }}\overline {\text{y}} {\text{ }}\overline {\text{z}} {\text{)}}$ helped by a complex transformation matrix ${\text{T}}$ to ensure $P{\text{l}}$ perpendicular to Z-axis (See Fig. 5(b)).

7 $${\mathop{\rm T}\nolimits} \, = \, {T_1}^{ - 1}\,*\,{T_2}^{ - 1}$$

Where T_1_ and T_2_are two rotation transformation matrix defined as follows

8 $${{\rm{T}}_1}{\rm{ = }}\left[ {\matrix{ 1 & 0 & 0 \cr 0 & {\sqrt {{{\cos }^2}\alpha + {{\cos }^2}\gamma } } & { - \cos \beta } \cr 0 & {\cos \beta } & {\sqrt {{{\cos }^2}\alpha + {{\cos }^2}\gamma } } \cr } } \right]$$

9 $${{\rm{T}}_2}{\rm{ = }}\left[ {\matrix{ {{{\cos \gamma } \over {\sqrt {{{\cos }^2}\alpha + {{\cos }^2}\gamma } }}} & 0 & {{{{\rm{ - }}\cos \alpha } \over {\sqrt {{{\cos }^2}\alpha + {{\cos }^2}\gamma } }}} \cr 0 & 1 & 0 \cr {{{\cos \alpha } \over {\sqrt {{{\cos }^2}\alpha + {{\cos }^2}\gamma } }}} & 0 & {{{\cos \gamma } \over {\sqrt {{{\cos }^2}\alpha + {{\cos }^2}\gamma } }}} \cr } } \right]$$

**Fig. 6:**
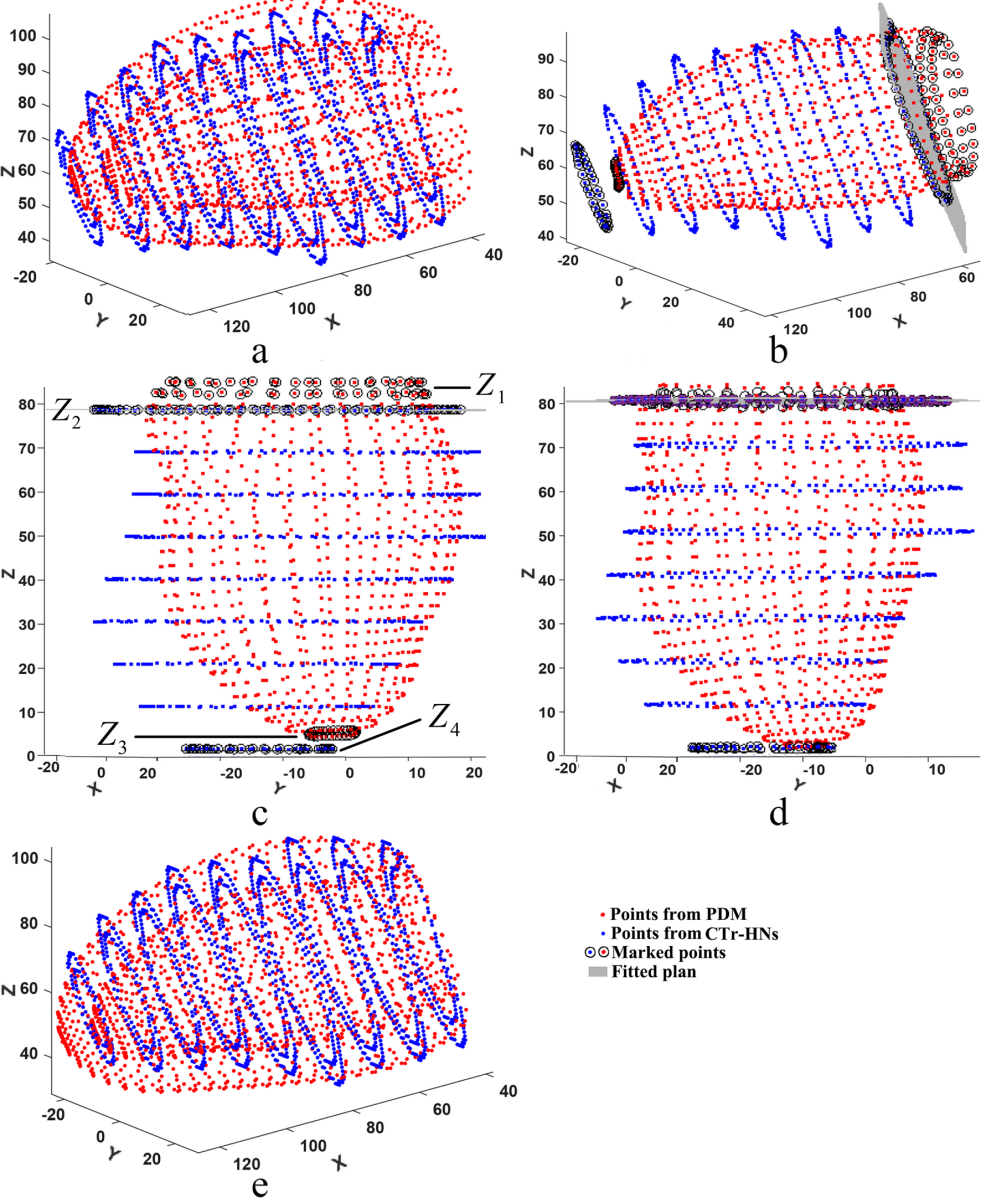
Initial shape refinement procedure. (**a**) Endocardial contour points from PDM and CTr-HNs at their original position, (**b**) Plane fitting for points from CTr-HNs, (**c**) Rotated endocardial contour points from PDM and CTr-HNs, (**d**) Stretched & aligned initial shape with CTr-HNs points, (**e**) Refined initial shape and CTr-HNs results in their original position. Points with black circles are adopted for plane fitting and transformation

Using the above technique, the endocardial contour points set from CTr-HNs in base slice is fitted and get a plane (See Fig. 6(a) and (b)). Then the fitted plane is rotated to be perpendicular to Z-axis (See Fig. 6(c)). Assume ${Z_1}$ and ${Z_4}$ are the average Z-axis values for the marked points set from PDM in base and apex slices respectively, ${Z_2}$ and ${Z_3}$ are their counterparts from CTr-HNs. A scale is applied to stretch points from PDM defined as follows:

10 $${\text{ratio}} = \frac{{{Z_2} - {Z_4}}}{{{Z_1} - {Z_3}}}$$

The points from PDM is stretched according to the ratio, and then aligned to the points from CTr-HNs (See Fig. 6(d)). A Procrustes analysis [32] is then employed to get a left ventricular model initialization in its original position (See Fig. 6). Once the CTr-HNs is trained, we can segment the blood pool and myocardium on SA CMR images, and get the initial endo- and epicardial contours. Two distance maps are constructed from the initial endo- and epicardial contours for SPASM segmentation, which were used in our previously published work [6, 7, 33]. The distance maps are helpful to eliminate the long range deviations between the target LV and the trained active shape model.

## Datasets

In this paper, UK BioBank dataset is adopted to train and test our CTr-HNs network. The UK Biobank encompasses short-axis and long-axis cine Cardiovascular Magnetic Resonance (CMR) images from 50,000 cardiac MRI cases, forming part of a large-scale, prospective, population-based study based in the United Kingdom. This initiative aims to investigate both genetic and non-genetic factors influencing a wide array of diseases. As part of this extensive research effort, CMR examinations are planned for an additional 100,000 participants, building upon the existing cohort of 500,000 middle-aged and older adults who have been recruited for comprehensive health studies.

The CTr-HNs network parameters are learned from short-axis (SA) view CMR images obtained from 700 subjects from the UK Biobank. In training phase, all MR images undergo a series of preprocessing steps, including slicing and the standardization of image dimensions. These images are then resized to a same size of 256 × 256 pixels through a combination of cropping and padding. The primary objective of the CTr-HNs network is to accurately distinguish between four classes: background, LV cavity, RV cavity and myocardium. Each case is accompanied by expert-drawn endocardial and epicardial contours, providing high-quality ground truth annotations essential for supervised learning.

After the CTr-HNs network is trained, more than 1200 cardiac MRI cases from CAP (Cardiac Atlas Project) dataset [34] are used for the SPASM segmentation. CAP is a resource for cardiac image data sharing and atlas-based shape analysis for population studies which can be web-accessible (http://www.cardiacatlas.org). The cases used in our work include two cohorts: asymptomatic volunteers (AV) and patients with myocardial infarction (MI). Manual contours were also provided by the Cardiac Atlas Project. Readers can refer to literature [35] for the detail about the imaging protocols of CAP.

## Results

### Evaluation of the method (segmentation accuracy measurement)

To validate the efficacy(performance) of the proposed model, we conducted the evaluation in two ways: 1) employing standard metrics for segmentation accuracy, such as the Dice coefficient, mean contour distance (MCD), and Hausdorff distance (HD95), and 2) utilizing clinically relevant measures derived from segmentations, including ventricular volume and mass. The Dice Coefficient serves as a metric to evaluate the overlap between the predicted segmentation and the ground truth. It ranges from 0 to 1, the closer the value is to 1, the higher overlap between the segmentation and ground truth. The mean contour distance quantifies the average distance between the contours derived from automatic segmentation and the ground truth, while the Hausdorff distance measures the maximum distance between the two segmentation contours. A lower the distance metric indicates a higher level of alignment between the two contours of the segmentation and the ground truth [36].

We have further conducted an evaluation of the accuracy of clinical metrics that are obtained from image segmentation. Specifically, we computed the left ventricular end-diastolic volume (LVEDV), end-systolic volume (LVESV), and myocardial mass (LVM) from the automated segmentations. These values were then compared to those derived from manual segmentations. The volumes were determined by summing the voxels corresponding to the relevant label class in the segmentation and multiplying by the volume per-voxel. As for the LV mass, it was calculated by multiplying the volume by a density of 1.05 g/mL [37].

### The CTr-HNs segmentation

To verify the performance of the proposed model, we compare the results between Bai and our CTr-HNs on a same test set of 600 subjects. Table 1 presents the experimental results of cardiac MRI image segmentation conducted on the UK Biobank dataset. The results demonstrate that our proposed approach achieves Dice coefficients (Dice), mean contour distances (MCD), and mean Hausdorff distances (HD95) of 0.95, 0.10, and 1.54 for the LV segmentation, respectively; 0.88, 0.13, and 1.94 for the myocardium segmentation; and 0.91, 0.24, and 3.25 for the RV segmentation. For the Dice, our method achieves outperformance compared to the Bai’s method in both LV and RV segmentation, while achieving comparable results in myocardium segmentation. Specifically, the Dice for LV cavity and RV cavity are improved by 0.01. For the MCD, all segmentation metrics showed significant improvement. Compared to Bai’s method, our method demonstrates a reduction in MCD by 0.94 for LV, 1.54 for RV, and 1.01 for myocardium. For the HD95, our proposed method also showed significant advantages, with achieving reductions of 1.62, 1.98, and 4.00 in the LV, myocardium and RV, respectively.

**Table 1:** Our proposed Segmentation Scheme on UK BioBank dataset

	Bai (4275 cases for training)			Proposed (700 cases for training)		
	Dice	MCD(mm)	HD95(mm)	Dice	MCD(mm)	HD95(mm)
LV cavity	0.94(0.04)	1.04(0.35)	3.16(0.98)	0.95(0.03)	0.10(0.09)	1.54(0.70)
LV myocardium	0.88(0.03)	1.14(0.40)	3.92(1.37)	0.88(0.03)	0.13(0.14)	1.94(0.86)
RV cavity	0.90(0.05)	1.78(0.70)	7.25(2.70)	0.91(0.03)	0.24(0.26)	3.25(1.84)
Avg	0.906(0.04)	1.32(0.48)	4.78(1.68)	0.913(0.03)	0.16(0.16)	2.24(1.13)

### The SPASM segmentation

To evaluate the precision of SPASM, we conducted a comparative analysis of point-to-surface (P2S) distances and the Dice score between the automated segmentation outcomes and the ground truth on CAP dataset.

To show the advantages of the proposed technique, P2S errors are calculated between ground truth and automatic shapes in Table 2. The overall P2S errors is 1.45 ± 0.51 mm for the proposed schema, while they are 2.11 ± 0.56 for SPASM adopted by Alba et al [19].

**Table 2:** Point to surface errors for the clinical cases (mm)

Method		Proposed	Alba’s (2018)
Diastolic phase	LV Endo	1.12 ± 0.39	1.64 ± 0.46
	LV Epi	1.32 ± 0.39	1.77 ± 0.47
	LV myocardium	1.11 ± 0.27	1.47 ± 0.31
Systolic phase	LV Endo	1.54 ± 0.70	2.21 ± 0.79
	LV Epi	1.97 ± 0.76	2.48 ± 0.33
	LV myocardium	1.62 ± 0.56	3.06 ± 1.02
	Overall	1.45 ± 0.51	2.11 ± 0.56

**Fig. 7:**
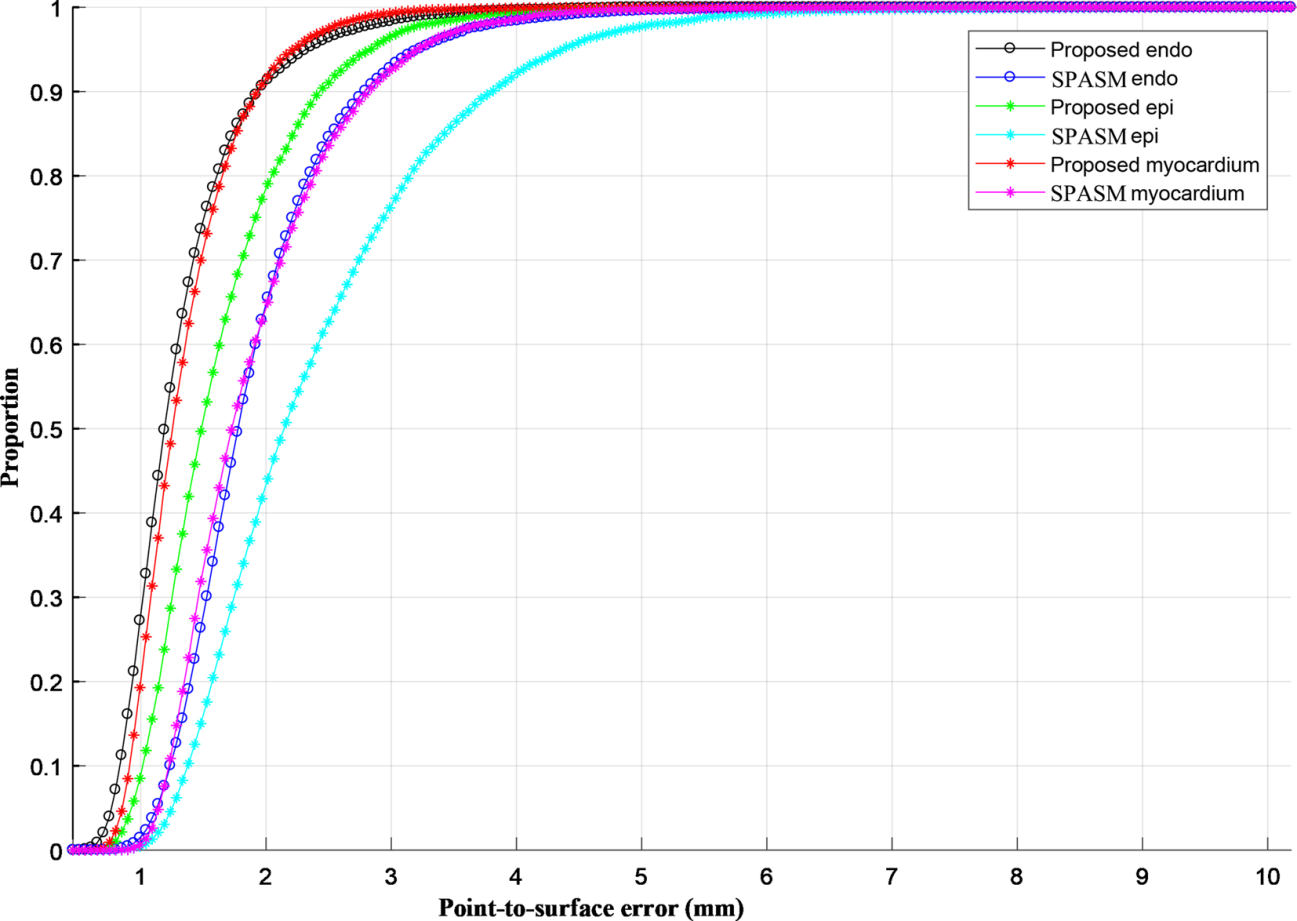
Cumulative point-to-surface error distribution curves for LV myocardium, endo- and epi-cardial for the results from the proposed and Alba’s (2018) [13]

The cumulative P2S error distribution curves are drawn in Fig. 7 for endocardium, epicardium and myocardium, which represent the cumulative percentages corresponding to the percentage of test cases for which the error is less than a specific value. In our schema, 90% of the P2S error are detected with a 1.9 mm for endocardium and myocardium, 2.4 mm for epicardium; they are greater than 2.8 mm for previous work respectively.

Table 3 shows the results of the clinical cases for two methods. In our proposed method, average Dice scores from endo- and epi-cardial contours are 0.87 and 0.91, respectively.

**Table 3:** Dice score for the clinical cases, ED (End-diastole), ES (End-systole)

		Endo			Epi		
		ED	ES	Average	ED	ES	Average
Dice	Proposed	0.88 ± 0.07	0.85 ± 0.09	0.87 ± 0.08	0.92 ± 0.06	0.90 ± 0.06	0.91 ± 0.06
	Alba’s (2018)	0.82 ± 0.09	0.66 ± 0.15	0.74 ± 0.12	0.79 ± 0.13	0.68 ± 0.15	0.74 ± 0.14

**Fig. 8:**
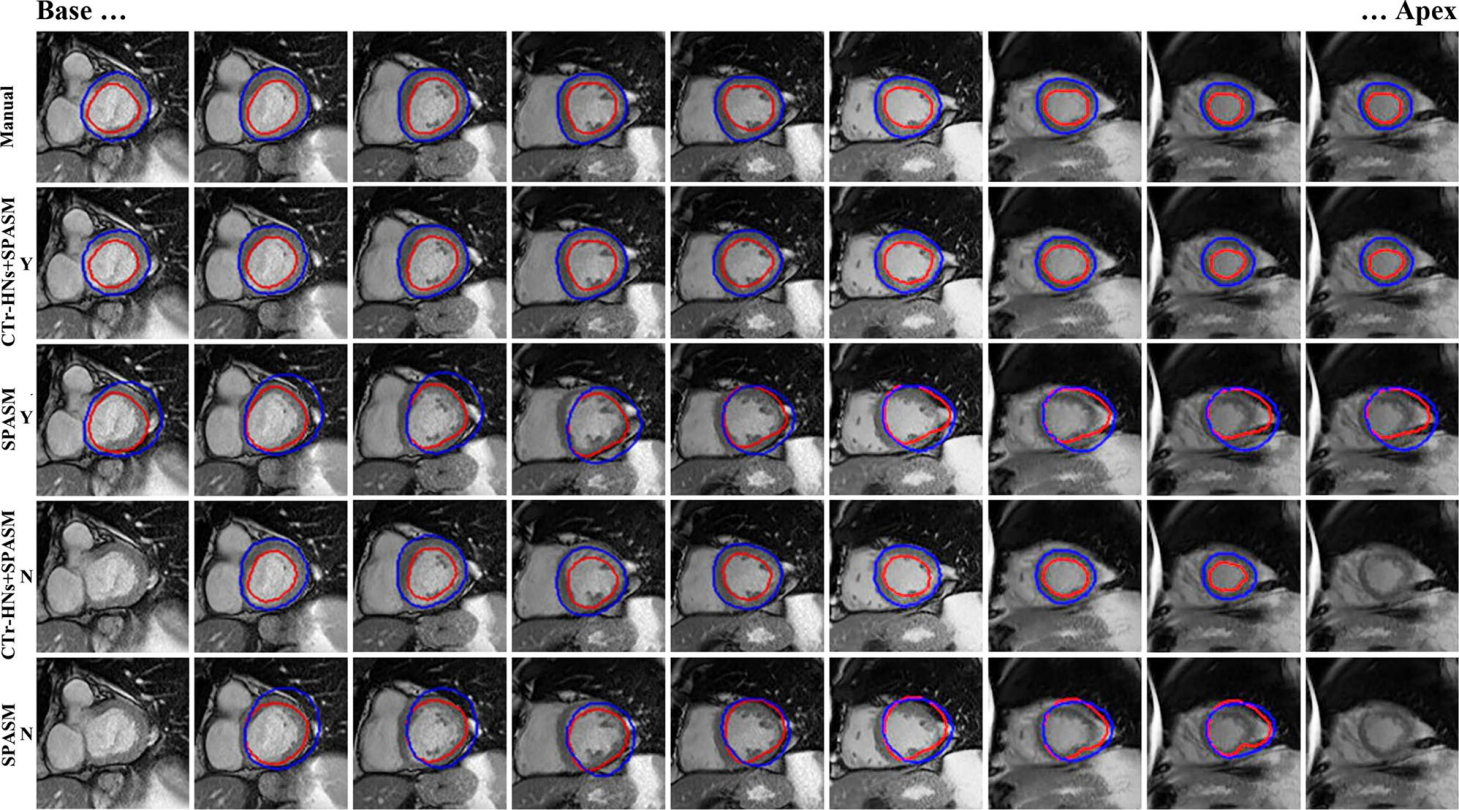
Short axis slice segmentation of one patient using different strategies (Y: using initial shape refinement; N: without initial shape refinement)

Figure 8 displays segmentation of one case using different methods with/without refined initial shape. It can be seen that base and apex slices may fail to be segmented for the case without optimized initial shape. However, only adopting initial shape refinement techniques may cause poor segmentation (see the third row images), and, hence, the coarse segment CTr-HNs result is employed to drive the contours to the correct location (see the second row images).

To evaluate cardiac function, clinic parameters are calculated for LVEDV, LVESV, LVSV, LVM and LVEF. In Table 4, it can been seen that the results from ours are close to those of experts.

**Table 4:** Cardiac functional indexes. MADif: Mean absolute difference

	From experts	Proposed	MADif
LVEDV (ml)	171.67 ± 49.39	166.33 ± 47.81	7.68
LVESV (ml)	97.92 ± 45.11	95.93 ± 43.67	6.28
LVSV (ml)	73.76 ± 28.13	70.41 ± 28.73	7.34
LVM (g)	148.74 ± 44.97	149.22 ± 44.23	8.30
LVEF (%)	44.17 ± 16.83	43.34 ± 17.71	3.22

**Fig. 9:**
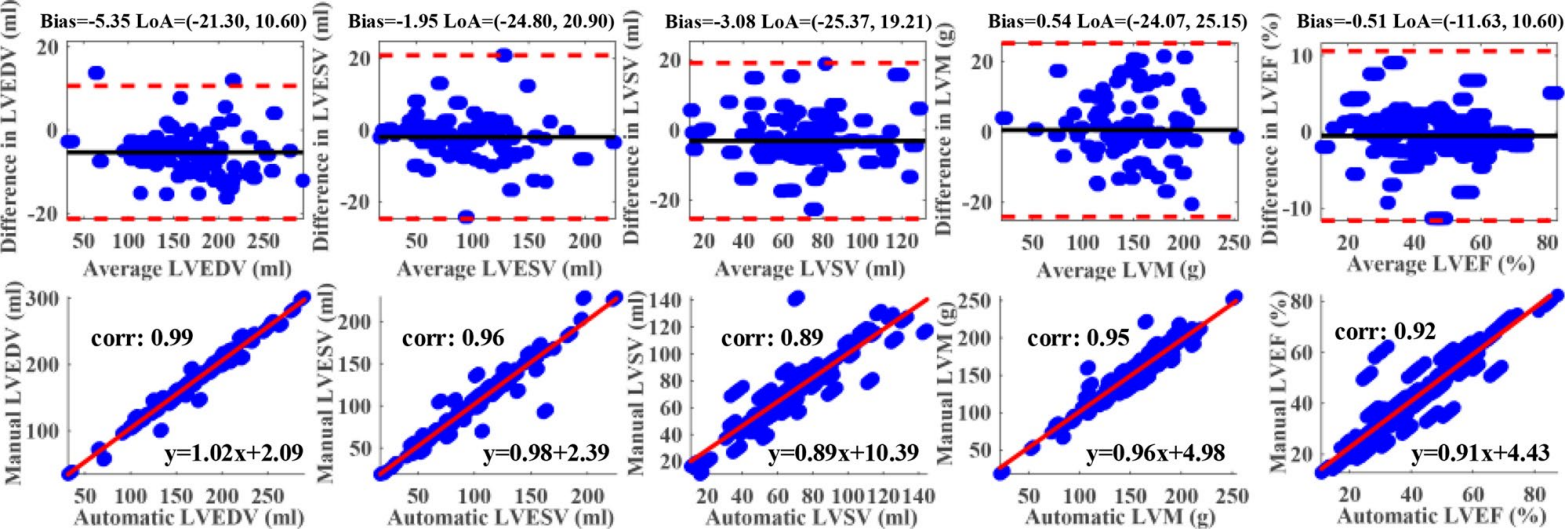
Plots of Bland–Altman and correlation of cardiac functional indexes between the manual and automatic results. In the top row, the mean difference (i.e., bias) and limits of agreement (LoA, i.e., ± 1.96 standard deviations from the mean) are denoted by black horizontal lines and the two red dashed lines respectively; while in the second row, the red lines represent correlation plots

To demonstrate whether the cardiac functional indexes derived from the ground truth align with those generated by our novel algorithm, we present Bland–Altman plots (displayed in the first row of Fig. 9) and correlation plots (shown in the second row of Fig. 9). These visualizations reveal a strongly match between our results and those from manual delineation. Correlations of cardiac indexes range from 0.89 to 0.99, demonstrating a strong relationship between manual and automatic methods.

## Discussion

This paper presents a fully automatic approach which can analyze cardiac MRI in large MRI studies. Our schema combines a deep learning neural network, an initial shape refinement algorithm, and a SPASM segmentation method. Different from other approaches, the initial shape derived from CTr-HNs results are rotated and scaled to cover all short slices using complex transformation techniques. Subsequently, the refined initial shape is adopted to obtain a three-dimensional LV segmentation based on a SPASM search.

In CTr-HNs segmentation experiment, we can observe that the standard deviation from ours is notably smaller in Dice metrics, which indicate a stable results and a certain level of performance improvement. Moreover, there is a significantly decrease on the MCD metric, demonstrating that CTr-HNs effectively optimize the segmentation boundaries of various tissues, thereby achieving more precise boundary localization. Additionally, the experimental results also reveal a significant improvement in HD95 on RV, further verifying that CTr-HNs can accurately capture the structural boundaries. These outcomes exhibit that our proposed method is capable of leveraging both global contextual information and local boundary features through the hybrid CNNs-Transformer architecture. Furthermore, by incorporating the edge feature guidance (EFG) module, it achieves more precise boundary information localization.

In SPASM, an initial estimate, denoted initial shape, describes the LV position. Considering the similar shapes and edge information between the endo- and epi-cardial contours, if initial shapes are in incorrect LV positions, failures with cardiac image segmentation using SPASM are inevitable. To get the initial shape for SPASM, point-sets registration method is used to align the points of mean shape to the counterparts from CTr-HNs. However, base or apex slice may be missing in the cover of the initial shape, this can be seen in Fig. 7 that poor results are obtained for SPASM when the initial shapes are failed to cover all short slices.

To overcome these difficulties, points from CTr-HNs in the base slice is fitted into a plane, and the fitted plane is rotated to be perpendicular to Z-axis. In the meantime, points from CTr-HNs and initial shape is rotated with the same angle. Note that the rotation is purposely designed, because the initial shape is easily to be scaled and moved in Z-axis direction only.

At last, CTr-HNs segmentation results are used in building distance maps and combined with an image intensity model to drive the initial shapes to the LV position. As a result, a 3D shape which represents an accurate segmentation for the LV is generated.

To confirm it is the same distribution of cardiac functional indexes from manual and automatic methods, Kolmogorov-Smirnov test analysis is adopted for the corresponding clinical parameters. It can be seen in the distribution plots a common distribution, common location and scale, similar distributional shapes.

A limitation of our framework lies in the heavy reliance of our algorithms on model-fitting techniques that utilize 3D active shapes to align cardiac contours across 2D imaging plane stacks. Consequently, the deep learning algorithm employed in this study is geared towards a trainable 2D segmentation model that integrates CNNs and Transformers as an encoder. This approach was chosen because the implemented SPASM method proves effective for increasingly sparse image datasets, encompassing various orientations and originating from different MRI acquisition protocols [27]. The incorporation of an update propagation scheme and a fuzzy inference system enabled application of SPASM to multi-protocol cardiac sparse data sets with a segmentation performance that is better than or comparable to other 3D model-based segmentation methods operating on a full data set with parallel image planes.

## Conclusion

This study introduces a hybrid schema that can automatically build initial shapes to cover all short slices for SPASM. Deep learning algorithms are employed not only for myocardial detection, but also to drive the shape model to the LV endo- and epi-cardial contours. Results indicate that our method can overcome technical difficulties and obtain robust segmentation for cardiac MRI studies with subvoxel accuracy. Our approach still can be improved in some aspects. For example, the detection of cardiac images with LVOT (left ventricular outflow) and how to use images with LVOT to optimize the initial shape and enhance segmentation using SPASM.

## Data Availability

This research has been conducted using the UK Biobank Resource under Applications 11350 and the Cardiac Atlas Project (CAP)，which can be web-accessible (http://www.cardiacatlas.org).

## Abbreviations

CNNs: Convolutional neuron networks
CTr-HNs: Integrated CNNs and Transformer for heart segmentation networks
LVEDV: Left ventricular End-diastolic Volume
LVEDV: Left ventricular End-diastolic Volume
LVM: Left ventricular mass
LVEF: Left ventricular ejection fraction
LVSV: Left ventricular stroke volume
ASM: Active shape model
LA: Long axis
SA: Short axis
CAP: Cardiac Atlas Project
CMR: Cardiac MRI
SPASM: Sparse active shape model
PDM: Point Distribution Model
IIM: Image intensity model
AV: Asymptomatic volunteers
ED: End-diastole
ES: End-systole
MI: Myocardial infarction
P2S: Point-to-surface
MCD: Mean contour distance
